# Clinical phenotypes and imaging evolution of hepatic portal venous gas under conservative management: a single-center case series using albumin and procalcitonin as prognostic stratification anchors

**DOI:** 10.3389/fmed.2026.1745715

**Published:** 2026-05-28

**Authors:** Biao Wang, Fengwei Yao, Weihua Fu

**Affiliations:** 1 Department of General Surgery, Tianjin Medical University General Hospital, Tianjin, China; 2 Department of Gastrointestinal Surgery, Renmin Hospital, Hubei University of Medicine, Shiyan, Hubei, China

**Keywords:** albumin, conservative management, hepatic portal venous gas, procalcitonin, prognosis, retrospective study

## Abstract

**Background:**

Hepatic portal venous gas (HPVG) is a rare but clinically significant radiologic finding associated with diverse intra-abdominal and systemic diseases. Once considered a fatal sign, its prognosis now varies widely due to advances in imaging and supportive care. This study aimed to characterize the clinical features, etiologies, imaging patterns, and prognostic factors of HPVG managed conservatively.

**Methods:**

A retrospective analysis was conducted on 16 patients diagnosed with HPVG by CT between January 2017 and June 2025 at Shiyan People’s Hospital, Hubei University of Medicine. Demographics, comorbidities, laboratory data, imaging findings, and outcomes were reviewed. Group comparisons employed the *t*-test, Mann–Whitney U, or chi-square test.

**Results:**

Among the 16 patients, 11 were male (68.8%), with a mean age of 61.1 ± 15.8 years. The major etiologies were intra-abdominal infection (31.3%), gastrointestinal bleeding (25%), intestinal obstruction (18.8%), and perforation (12.5%). All received conservative management, with an in-hospital mortality rate of 62.5%. Non-survivors had significantly lower serum albumin and higher procalcitonin (PCT) levels, while brain natriuretic peptide (BNP) showed weak association with mortality. Other demographic and biochemical variables showed no significant differences.

**Conclusion:**

In conservatively treated HPVG, hypoalbuminemia and elevated PCT were strongly associated with mortality. Imaging follow-up demonstrated complete gas resolution in several patients after control of the underlying disease, indicating HPVG represents a radiologic manifestation rather than a disease entity. Combined evaluation of imaging dynamics and biomarkers may aid early risk stratification and guide individualized management. Further multicenter prospective studies are warranted to validate these findings.

## 1 Background

Hepatic portal venous gas (HPVG) is a rare but potentially life-threatening radiologic sign characterized by the presence of gas within the portal venous system. Since its first description by Wolfe and Evans in 1955, HPVG has traditionally been regarded as a surgical emergency, with early reports citing mortality rates exceeding 70% (1–3). With the widespread use of high-resolution computed tomography (CT), the detection rate of HPVG has increased substantially. It is now recognized in a broad range of clinical contexts, from life-threatening bowel ischemia to benign or iatrogenic causes such as endoscopic manipulation, postoperative states, or infectious enteritis (4, 5). From an imaging perspective, a single-center database encompassing 134,800 abdominal ultrasound (US) examinations demonstrated that HPVG is rare but not always associated with bowel ischemia, and in a small subset of cases, US may even be more sensitive than CT (6).

Meanwhile, the etiologic spectrum of HPVG has expanded from the classical ischemic or necrotic intestinal conditions to benign, iatrogenic, and toxicologic scenarios. Some studies have suggested that most patients present with severe intra-abdominal pathology requiring urgent surgical intervention (7). However, transient and self-limited HPVG has also been reported following endoscopic or barium-related procedures in patients with inflammatory bowel disease (IBD) (8). Literature reviews indicate that these cases often respond well to conservative treatment and have much lower mortality rates than historically reported. In contrast, HPVG observed in toxic exposures such as glyphosate ingestion or after rapid enteral nutrition infusion may signal more profound systemic injury and demand closer monitoring and decision-making (9). Therefore, HPVG should be interpreted as a radiologic clue reflecting an underlying pathologic process rather than a direct indication for surgery.

Recent studies have reshaped the conventional perception of HPVG. Multiple retrospective analyses and systematic reviews have shown that prognosis primarily depends on the underlying etiology and the presence of high-risk imaging features rather than the gas itself (3, 10). Some researchers have proposed a dual-dimensional “imaging–clinical” stratification model to differentiate patients requiring emergency surgery from those suitable for conservative management. For instance, Gonda et al. (4) reported that patients without peritoneal irritation, hypotension, or CT evidence of bowel ischemia could often be managed conservatively (4), while Wei et al. (3) identified thrombocytopenia, neutrophilia, hypertension, and peritonitis as potential indicators favoring surgical intervention (3).

Despite these advances, existing studies remain limited by small sample sizes, inconsistent diagnostic criteria, and insufficient imaging–clinical correlation analyses. In Asia, particularly in China, systematic data from tertiary hospitals on HPVG remain scarce (11, 12). Clarifying the clinical spectrum and outcomes of HPVG in different populations is essential for refining early recognition and management strategies.

Based on these considerations, the present study focused on (1) the clinical characteristics and imaging features of HPVG; (2) management strategies across heterogeneous etiologies; and (3) biomarkers for early risk stratification under conservative treatment. We retrospectively analyzed 16 patients diagnosed with HPVG at Shiyan People’s Hospital, affiliated with Hubei University of Medicine, between 2017 and 2025, aiming to enhance the comprehensive understanding of HPVG and provide insights for clinical decision-making.

## 2 Materials and methods

### 2.1 Study design and ethical approval

This was a retrospective case series conducted at Shiyan People’s Hospital, a tertiary teaching hospital affiliated with Hubei University of Medicine. All cases diagnosed with hepatic portal venous gas (HPVG) by computed tomography (CT) between January 2017 and June 2025 were retrieved from the institutional radiology database. Cases were screened through the electronic medical record system and radiology information system of our hospital. Between January 2017 and June 2025, 16 patients with CT-confirmed HPVG were identified. No surgically managed HPVG cases were found during the same period in our institutional database; therefore, all eligible cases in this series received conservative treatment. This treatment pattern reflects the real-world practice of our institution rather than investigator-driven selection. The study adhered to the Declaration of Helsinki and relevant ethical guidelines and was approved by the Ethics Committee of Shiyan People’s Hospital (Approval No. SYRMYY-2025-120). Given the retrospective design, all data were extracted from the hospital information system without direct patient contact or intervention. Personal identifiers were re-coded prior to analysis, and the requirement for informed consent was waived by the Ethics Committee.

### 2.2 Patient selection and diagnostic criteria

All patients diagnosed with HPVG based on CT imaging between January 2017 and June 2025 were retrospectively reviewed. Eligible cases were identified through the institutional radiology information system. To ensure diagnostic consistency, the definition of HPVG followed previously published radiological criteria (3).

HPVG was confirmed when gas was clearly visible within the intrahepatic portal venous branches on CT, typically presenting as branched or linear low-attenuation areas extending toward the hepatic periphery. Each case was independently reviewed by two senior radiologists with more than 10 years of experience in abdominal imaging, and disagreements were resolved by consensus. In the present study, albumin and PCT were analyzed as continuous variables. For clinical reference, serum albumin < 35 g/L was considered below the normal range and PCT > 0.5 ng/mL was considered elevated according to our institutional laboratory standards.

### 2.3 Data collection

Patient data were extracted from the electronic medical record system and organized using a standardized case report form (CRF). Four domains of information were collected for each case:

1. Demographics: age and sex;
2. Clinical characteristics: admission diagnosis, etiology, and comorbidities (including hypertension, diabetes, cardiovascular disease, malignancy, or end-stage renal disease);
3. Laboratory parameters: white blood cell and neutrophil counts, platelets, hemoglobin, liver function tests (ALT, AST, total bilirubin), renal function (serum creatinine, blood urea nitrogen), and inflammatory markers (C-reactive protein and procalcitonin, if available);
4. Imaging and clinical outcomes: CT features (extent and distribution of HPVG, presence of pneumatosis intestinalis, mesenteric venous gas, free intraperitoneal air, or ascites) and patient outcome (survival or death).

Data extraction was performed independently by two investigators and cross-checked for accuracy. Any discrepancies were resolved by review of the original medical records and imaging reports by a senior physician. To minimize bias, all data were anonymized prior to analysis in accordance with institutional data-protection regulations.

### 2.4 Statistical analysis

All analyses were performed using SPSS version 26.0 (IBM Corp., Armonk, NY, USA). The Shapiro–Wilk test was applied to assess the normality of continuous variables. Normally distributed variables were expressed as mean ± standard deviation (SD), and non-normally distributed variables as median with interquartile range [M (IQR)]. Categorical variables were summarized as counts and percentages (*n*, %).

Comparisons between survivors and non-survivors were conducted as follows: independent-samples *t*-test for normally distributed continuous variables, Mann–Whitney U test for non-normally distributed variables, and chi-square or Fisher’s exact test for categorical variables. All tests were two-tailed, and *P* < 0.05 was considered statistically significant. Missing data were handled by case-wise deletion without imputation.

Results were presented in tables and figures to illustrate patient characteristics, imaging findings, and outcomes. Statistical methods were selected according to data distribution and variable type, following the STROBE reporting guidelines for observational studies. All analyses were independently reviewed by a second statistician to ensure reproducibility.

## 3 Results

### 3.1 Baseline characteristics

A total of 16 patients with CT-confirmed hepatic portal venous gas (HPVG) were included (male 11 [68.75%], female 5 [31.25%]); the mean age was 61.12 ± 15.81 years. Compared with standard reference ranges, laboratory profiles suggested multisystem derangements consistent with systemic inflammation and organ dysfunction. White blood cell count was elevated (15.23 × 10^9^/L), C-reactive protein was markedly increased (125.15 mg/L), and the median procalcitonin (PCT) level was 4.65 ng/mL, collectively indicating a substantial bacterial/inflammatory burden.

Liver enzymes showed mild-to-moderate hepatocellular injury (ALT 24.5 U/L, AST 34.95 U/L). Cardiac biomarkers suggested myocardial stress or dysfunction, with a markedly elevated median B-type natriuretic peptide (BNP) (4,423.5 [428.8–14,507.25] pg/mL) and increased cardiac troponin T (cTnT) (0.03 [0–0.20] ng/mL).

Renal indices were impaired, with raised serum creatinine (112.3 [72.38–186.1] μmol/L) and blood urea nitrogen (11.69 mmol/L). Serum albumin was notably reduced (29.34 g/L), consistent with malnutrition and/or decreased hepatic synthetic function.

Taken together, patients with HPVG in this cohort commonly exhibited pronounced systemic inflammation, multi-organ dysfunction, and diminished metabolic reserve—features that are plausibly linked to adverse outcomes. Detailed distributions are provided in Table 1.

**TABLE 1:** Baseline characteristics of patients with hepatic portal venous gas (HPVG).

Variables	Values	Normal values
Total	16	–
Gender (male/female)	11 (68.75%)/5 (31.25%)	–
Age, (years)	61.12 ± 15.81	–
White blood cell count (×10^9^/L)	15.23 ± 5.23	4.0–10.0
Neutrophils (×10^9^/L)	88.05 (83.1–92.45)	40–75
Red blood cell count (×10^12^/L)	3.70 ± 0.85	Male: 4.3–5.8; Female: 3.8–5.1
Platelet count (×10^9^/L)	211.75 ± 103.28	100–300
Hemoglobin (g/L)	107.81 ± 28.32	Male: 130–175; Female: 115–150
C-reactive protein (mg/L)	125.15 ± 86.51	< 10
Procalcitonin (ng/mL)	4.65 (0.3–8.78)	< 0.05
Alanine aminotransferase (U/L)	24.5 (12.75–53.0)	9–50
B-type natriuretic peptide (pg/mL)	4,423.5 (428.8–14,507.25)	< 100
Serum creatinine (μmol/L)	112.3 (72.38–186.1)	Male: 57–111; Female: 44–97
Cardiac troponin T (ng/mL)	0.03 (0–0.20)	< 0.014
Potassium (mmol/L)	3.96 ± 0.52	3.5–5.5
Sodium (mmol/L)	140.11 ± 5.99	135–145
Chloride (mmol/L)	101.50 ± 4.93	98–107
Calcium (mmol/L)	1.98 (1.85–2.30)	2.1–2.6
Blood urea nitrogen (mmol/L)	11.69 ± 6.81	2.9–8.2
Blood glucose (mmol/L)	6.87 (5.29–7.68)	3.9–6.1
Serum albumin (g/L)	29.34 ± 8.62	35–50
Aspartate aminotransferase (U/L)	34.95 (22–109)	15–40

Continuous variables are presented as mean ± SD or median (IQR), as appropriate; categorical variables as *n* (%). Reference ranges are provided in the rightmost column.

### 3.2 Etiologies and comorbidities

Etiologic analysis showed that intra-abdominal infection was the most frequent cause of HPVG (5/16, 31.25%), followed by gastrointestinal bleeding (4/16, 25.0%) and unknown cause (4/16, 25.0%). Less common etiologies included intestinal obstruction (3/16, 18.75%), gastrointestinal perforation (2/16, 12.5%), intra-abdominal tumor (1/16, 6.25%), and high-fall trauma (1/16, 6.25%).

Regarding comorbidities, hypertension was most prevalent (6/16, 37.5%), followed by coronary heart disease (3/16, 18.75%), diabetes mellitus (2/16, 12.5%), malignancy (2/16, 12.5%), and end-stage renal disease (2/16, 12.5%).

Collectively, these data suggest that HPVG in our cohort most often occurred in the setting of severe intra-abdominal infection or acute gastrointestinal emergencies, in patients with a substantial comorbidity burden and generally compromised systemic condition. Detailed distributions are provided in Table 2.

**TABLE 2:** Etiologies and comorbidities among patients with HPVG.

Comorbidities	*n*[%]
Diabetes	2 (12.5%)
Hypertension	6 (37.5%)
Coronary heart disease	3 (18.75%)
Malignancy	2 (12.5%)
End-stage renal disease	2 (12.5%)
Etiologies of HPVG	–
Gastrointestinal perforation	2 (12.5%)
Intra-abdominal infection	5 (31.25%)
Gastrointestinal bleeding	4 (25%)
Unknown cause (abdominal pain)	4 (25%)
Intestinal obstruction	3 (18.75%)
Trauma: high-fall	1 (6.25%)
Intra-abdominal tumor	1 (6.25%)

Etiologies were adjudicated by imaging/endoscopy/MDT discussion; comorbidities were defined by documented medical history. “Unknown cause” indicates no clear precipitating factor identified at discharge.

### 3.3 Radiological features

**FIGURE 1:**
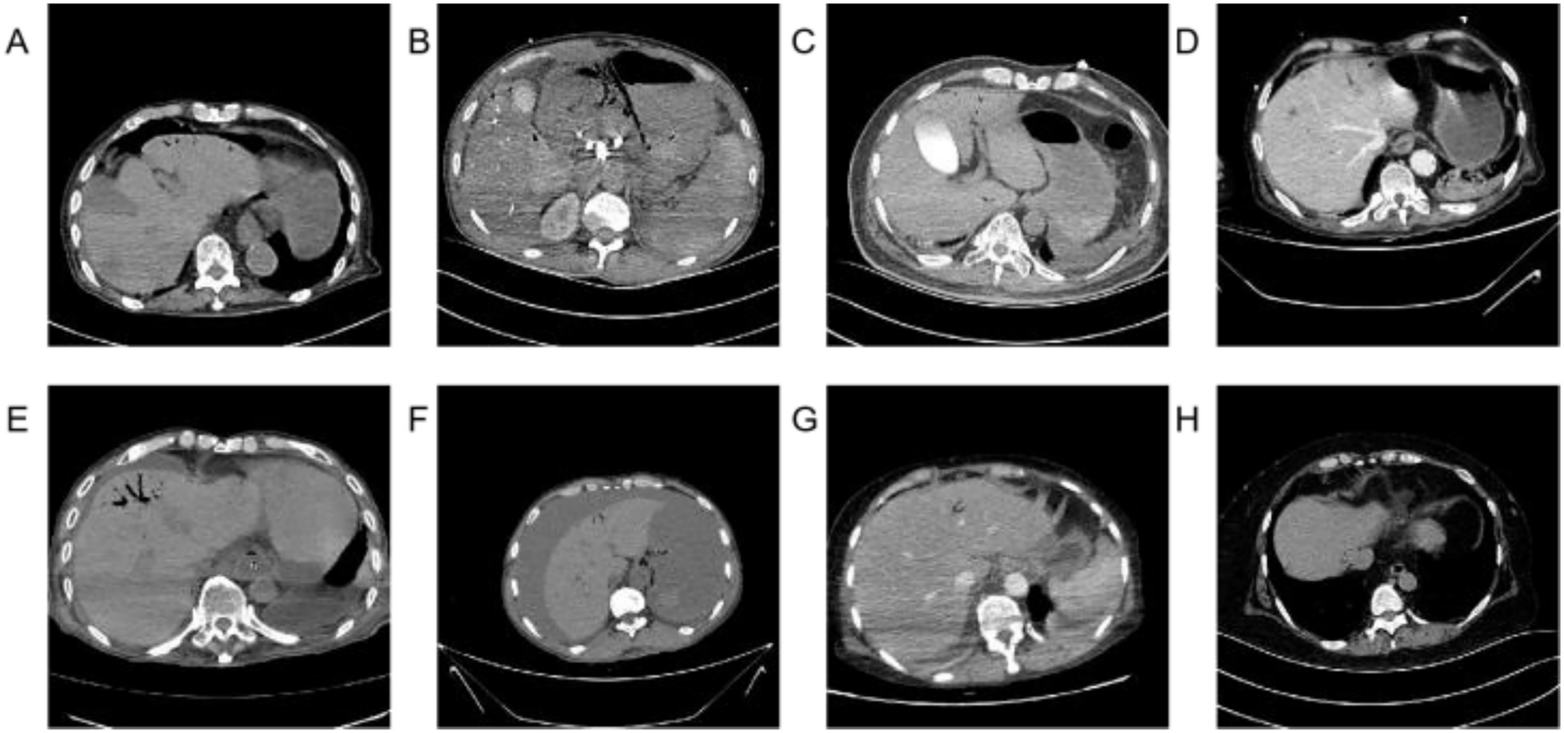
Representative contrast-enhanced CT images of HPVG confined to a single hepatic segment **(A–H)**. HPVG appears as peripheral branching/punctate hypoattenuation along portal venous branches.

**FIGURE 2:**
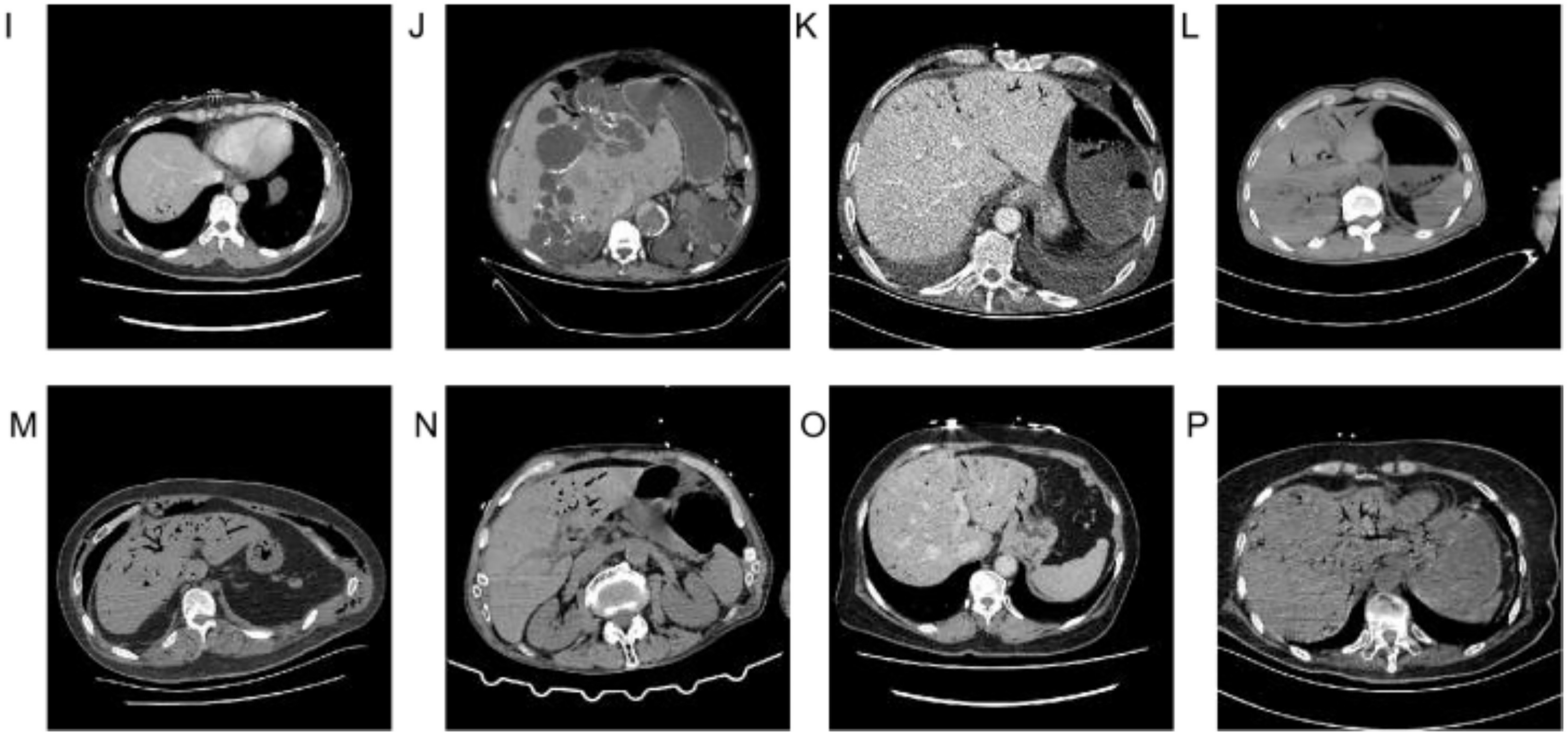
Representative contrast-enhanced CT images of multi-segment HPVG **(I–P)**, with gas extending to two or more hepatic segments and clustering toward the subcapsular or hilar regions.

Figures 1, 2 illustrate contrast-enhanced CT findings from 16 representative patients with hepatic portal venous gas (HPVG). In Figures 1A–H, gas is confined to a single hepatic segment; in Figures 2I–P, gas involves two or more segments. On CT, HPVG typically appears as peripheral, branching or punctate low-attenuation foci tracking along the intrahepatic portal venous branches, occasionally clustering around the hepatic surface or hilum. The extent and distribution varied substantially among patients and appeared to correlate with the underlying etiology and clinical severity.

**FIGURE 3:**
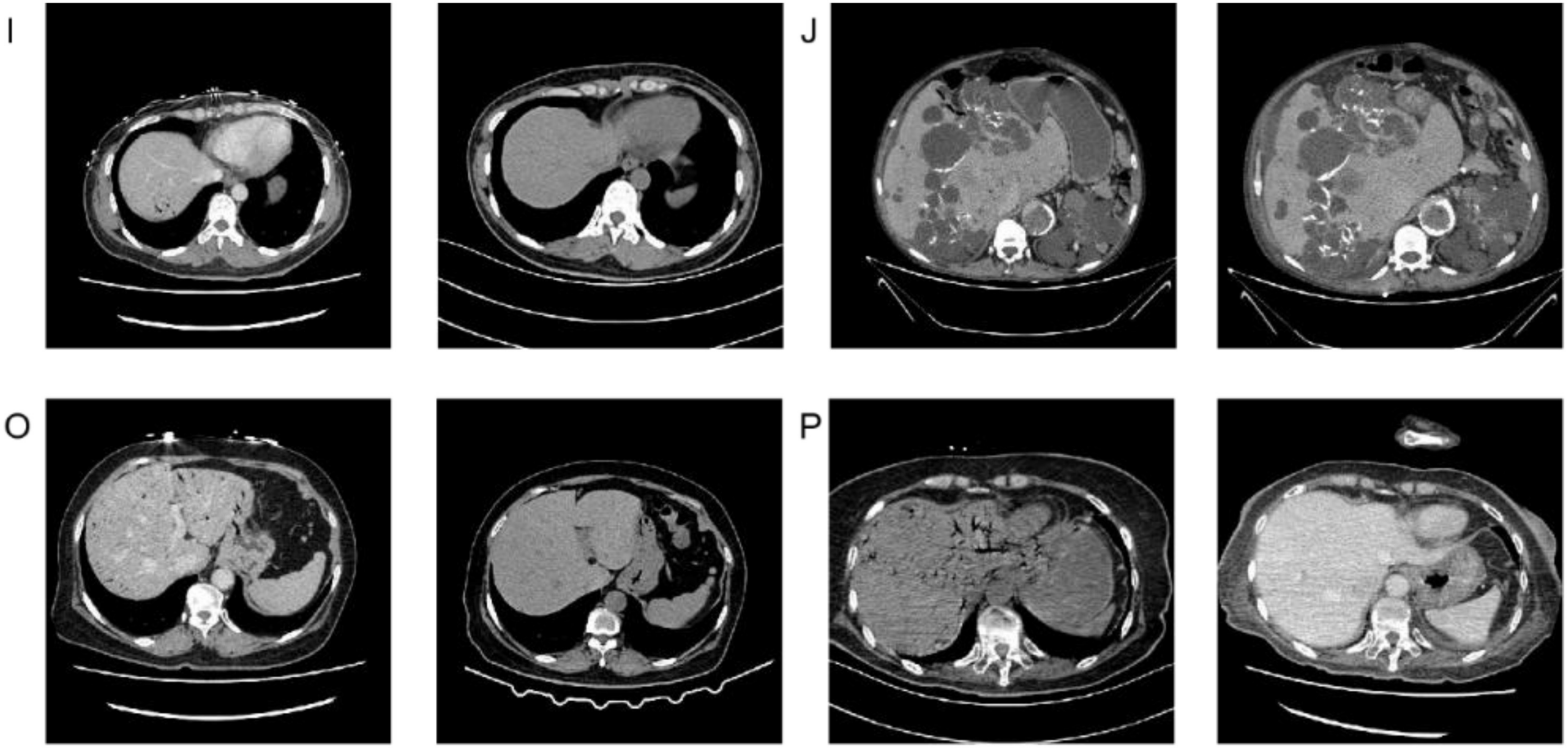
Case vignettes demonstrating temporal evolution of HPVG under conservative treatment. **(I)** HPVG related to gastroduodenal inflammation, complete resolution at day 6; **(J)** HPVG in a patient on chronic hemodialysis with ascites, complete resolution at day 3 after drainage and antibiotics; **(O)** Extensive HPVG resolving by day 10 on ultrasound; **(P)** HPVG with suspected ischemic colitis, complete CT resolution at day 84 after comprehensive medical therapy.

Because of the retrospective nature of the study and the heterogeneity of clinical conditions, imaging follow-up was not performed according to a fixed protocol. Instead, repeat CT or ultrasound was obtained on an individualized basis according to symptom evolution, treatment response, and routine clinical judgment. Figure 3 presents four illustrative cases. Figure 3I developed HPVG in the context of duodenitis and chronic erosive gastritis—gas completely resolved after 6 days of antimicrobial therapy. Figure 3J, with long-term hemodialysis and ascites, underwent ultrasound-guided drainage, dialysis optimization, and antibiotics, achieving complete radiologic resolution within 3 days. Figure 3O exhibited more extensive, segment-multifocal HPVG on presentation; follow-up ultrasound at 10 days showed full resolution after conservative care. Figure 3P, who had suspected ischemic colitis with mesenteric edema and gallstones, improved with anti-infective, hemostatic, and microbiota-modulating therapy, and CT at day 84 confirmed complete resolution.

Taken together, these observations indicate that HPVG most commonly manifests as peripheral, branching lucencies that may be segment-limited or multi-segmental. In the majority of cases, gas resolved on follow-up once the precipitating condition was treated, supporting the interpretation of HPVG as an imaging signal of the underlying pathology rather than a stand-alone therapeutic indication. No validated quantitative grading system for HPVG extent was applied in this study. Radiologic extent was assessed descriptively according to CT distribution, including segment-limited versus multi-segment involvement.

### 3.4 Treatment and outcomes

All 16 patients with hepatic portal venous gas (HPVG) in this study received conservative management without surgical intervention. The primary therapeutic strategies included broad-spectrum antibiotics, gastrointestinal decompression, fluid resuscitation, electrolyte correction, oxygen therapy, and supportive treatment for hepatic, renal, and cardiac function. Several patients demonstrated clinical and radiological improvement after treatment, with follow-up CT scans showing complete or partial gas resolution. However, the majority deteriorated due to severe infection or multi-organ failure.

Among all patients, 6 (37.5%) survived and were discharged in stable condition, while 10 (62.5%) died during hospitalization. The leading causes of death were sepsis and multiple organ dysfunction syndrome (MODS).

Comparative analysis between survivors and non-survivors revealed that serum albumin was significantly lower in the non-survival group (23.55 ± 4.10 vs. 39.00 ± 3.70 g/L, *P* < 0.001), while procalcitonin (PCT) levels were markedly higher (7.44 [5.57–10.71] vs. 0.18 [0.08–0.30] ng/mL, *P* < 0.001). Additionally, BNP levels were elevated in non-survivors compared to survivors (10,814.5 [2,370.5–23,435.3] vs. 398.8 [93.8–1,202.2] pg/mL, *P* = 0.050). Blood urea nitrogen (BUN) and AST levels also tended to be higher in the non-survival group, although without statistical significance (*P* = 0.079 and *P* = 0.057, respectively). Other laboratory parameters—including WBC, CRP, hepatic and renal function indices, electrolytes, and glucose—did not differ significantly between the two groups (*P* > 0.05).

There were no significant differences in comorbidities (e.g., diabetes, hypertension, coronary artery disease, malignancy, or end-stage renal disease) or admission etiologies (e.g., abdominal infection, gastrointestinal bleeding, intestinal obstruction, or unexplained abdominal pain) between survivors and non-survivors (all *P* > 0.05).

Overall, these findings suggest that hypoalbuminemia and elevated PCT levels may serve as potential risk factors for poor prognosis in HPVG patients, underscoring the crucial role of infection control and nutritional status in clinical outcomes. Nevertheless, due to the small sample size (*n* = 16), these results should be interpreted with caution. Detailed data are presented in Table 3.

**TABLE 3:** Comparison of clinical, laboratory, and outcome variables between survivors and non-survivors.

Variables	Total (*n* = 16)	Alive (*n* = 6)	Death (*n* = 10)	*P*
Age, mean ± SD	61.12 ± 15.82	52.83 ± 15.72	66.10 ± 14.36	0.106
WBC, mean ± SD	15.23 ± 5.23	15.15 ± 7.58	15.27 ± 3.69	0.968
RBC, mean ± SD	3.70 ± 0.85	3.72 ± 0.56	3.69 ± 1.02	0.958
PLT, mean ± SD	211.75 ± 103.28	250.17 ± 101.63	188.70 ± 102.30	0.263
HGB, mean ± SD	107.81 ± 28.32	105.17 ± 26.04	109.40 ± 30.86	0.783
CRP, mean ± SD	125.15 ± 86.51	94.75 ± 76.80	143.39 ± 90.61	0.292
K, mean ± SD	3.96 ± 0.52	3.68 ± 0.28	4.12 ± 0.57	0.101
Na, mean ± SD	140.11 ± 5.99	140.72 ± 3.80	139.74 ± 7.17	0.764
Cl, mean ± SD	101.50 ± 4.93	102.18 ± 3.57	101.09 ± 5.74	0.683
Bun, mean ± SD	11.69 ± 6.81	7.84 ± 3.03	13.99 ± 7.51	0.079
Albumin, mean ± SD	29.34 ± 8.62	39.00 ± 3.70	23.55 ± 4.10	< 0.001
N, M (Q_1_, Q_3_)	88.05 (83.10, 92.45)	86.55 (81.75, 87.53)	92.10 (84.62, 93.42)	0.073
PCT, M (Q_1_, Q_3_)	4.65 (0.30, 8.78)	0.18 (0.08, 0.30)	7.44 (5.57, 10.71)	< 0.001
ALT, M (Q_1_, Q_3_)	24.50 (12.75, 53.00)	13.00 (12.00, 37.40)	33.00 (24.00, 74.75)	0.143
BNP, M (Q_1_, Q_3_)	4,423.50 (428.80, 14,507.25)	398.80 (93.83, 1,202.20)	10,814.50 (2,370.50, 23,435.25)	0.05
Creatinine, M (Q_1_, Q_3_)	112.30 (72.38, 186.10)	72.15 (64.88, 155.33)	143.90 (96.10, 186.10)	0.254
Ctnt, M (Q_1_, Q_3_)	0.03 (0.00, 0.20)	0.00 (0.00, 0.04)	0.11 (0.01, 0.54)	0.112
Ca, M (Q_1_, Q_3_)	1.98 (1.85, 2.30)	2.17 (2.00, 2.38)	1.88 (1.79, 2.13)	0.174
Glucose, M (Q_1_, Q_3_)	6.87 (5.29, 7.68)	6.43 (5.93, 7.46)	6.98 (3.80, 7.70)	0.914
AST, M (Q_1_, Q_3_)	34.95 (22.00, 109.00)	24.00 (19.25, 30.25)	88.00 (28.25, 247.00)	0.057
Gender, *n* (%)				0.299
F	5 (31.25)	3 (50.00)	2 (20.00)	
M	11 (68.75)	3 (50.00)	8 (80.00)	
Diabetes, *n* (%)				>0.99
No	14 (87.50)	5 (83.33)	9 (90.00)	
Yes	2 (12.50)	1 (16.67)	1 (10.00)	
Hypertension, *n* (%)				>0.99
No	10 (62.50)	4 (66.67)	6 (60.00)	
Yes	6 (37.50)	2 (33.33)	4 (40.00)	
Coronary heart disease, *n* (%)				>0.99
No	13 (81.25)	5 (83.33)	8 (80.00)	
Yes	3 (18.75)	1 (16.67)	2 (20.00)	
Malignant tumor, *n* (%)				>0.99
No	14 (87.50)	5 (83.33)	9 (90.00)	
Yes	2 (12.50)	1 (16.67)	1 (10.00)	
End-stage renal disease, *n* (%)				>0.99
No	14 (87.50)	5 (83.33)	9 (90.00)	
Yes	2 (12.50)	1 (16.67)	1 (10.00)	
Gastrointestinal perforation, *n* (%)				0.5
No	14 (87.50)	6 (100.00)	8 (80.00)	
Yes	2 (12.50)	0 (0.00)	2 (20.00)	
Intra-abdominal infection, *n* (%)				0.588
No	11 (68.75)	5 (83.33)	6 (60.00)	
Yes	5 (31.25)	1 (16.67)	4 (40.00)	
Gastrointestinal bleeding, *n* (%)				0.604
No	12 (75.00)	4 (66.67)	8 (80.00)	
Yes	4 (25.00)	2 (33.33)	2 (20.00)	
Unknown cause (abdominal pain), *n* (%)				0.604
No	12 (75.00)	4 (66.67)	8 (80.00)	
Yes	4 (25.00)	2 (33.33)	2 (20.00)	
Intestinal obstruction, *n* (%)				0.25
No	13 (81.25)	6 (100.00)	7 (70.00)	
Yes	3 (18.75)	0 (0.00)	3 (30.00)	
Trauma: high-fall, *n* (%)				0.375
No	15 (93.75)	5 (83.33)	10 (100.00)	
Yes	1 (6.25)	1 (16.67)	0 (0.00)	
Intra-abdominal tumor, *n* (%)				>0.99
No	15 (93.75)	6 (100.00)	9 (90.00)	
Yes	1 (6.25)	0 (0.00)	1 (10.00)	

Data are shown as mean ± SD, median (IQR), or *n* (%). Group comparisons used independent-samples *t*-test, Mann–Whitney U test, or Fisher’s exact/χ^2^ test as appropriate. *P*-values are two-tailed; bold indicates *P* < 0.05.

## 4 Discussion

In this retrospective series of 16 patients with hepatic portal venous gas (HPVG) managed conservatively, the in-hospital mortality was 62.5%. Serum albumin and procalcitonin (PCT) emerged as salient prognostic indicators: non-survivors had significantly lower albumin and higher PCT, whereas brain natriuretic peptide (BNP) exhibited only borderline differences between groups. No significant between-group differences were observed in age, comorbidities, or other laboratory indices. BNP was included as an exploratory marker of hemodynamic stress and possible cardiac dysfunction in critically ill patients. However, because its association with mortality was only borderline in statistical significance in our cohort, we did not consider it a primary prognostic finding and therefore did not further emphasize it in the discussion. The cohort had a mean age of 61 years and was predominantly male (68.75%). Laboratory profiles frequently showed leukocytosis/neutrophilia and marked elevations in CRP and PCT, indicating a systemic inflammatory or infectious milieu. Mild-to-moderate anemia and hypoalbuminemia were also common, consistent with malnutrition and chronic inflammation.

With respect to etiology, intra-abdominal infection (31.25%) was most frequent, followed by gastrointestinal bleeding (25%), intestinal obstruction (18.75%), and gastrointestinal perforation (12.5%). Common comorbidities included hypertension (37.5%), diabetes (12.5%), and coronary heart disease (18.75%). Prior reports suggest that gas may enter the portal system through increased intraluminal pressure, mucosal injury, or translocation of gas-forming organisms across a compromised intestinal wall (13–15). Chronic conditions such as diabetes and cardiovascular disease may exacerbate mesenteric microcirculatory dysfunction and mucosal permeability, facilitating HPVG. In line with the literature, abdominal infection and intestinal pathology predominated in our cohort, reinforcing the view that HPVG primarily reflects the severity of underlying intra-abdominal disease.

The in-hospital mortality observed here falls within the broad range reported by prior studies (approximately 30–75%), which varies according to etiology and management strategy (16). Consistent with earlier work, the prognosis of HPVG is largely determined by the underlying disease burden and the intensity of systemic inflammation, rather than by the presence of gas itself. Our findings further underscore the early warning value of albumin and PCT.

Hypoalbuminemia integrates malnutrition, capillary leak, and systemic inflammation in the critically ill. Reduced albumin may reflect impaired hepatic synthesis and inflammation-driven proteolysis, thereby lowering plasma oncotic pressure, promoting bowel wall interstitial edema and mucosal ischemia, and facilitating bacterial translocation with escalation of the inflammatory cascade (17). Prior reports emphasize that outcomes in HPVG are determined chiefly by the underlying disease and the systemic inflammatory response, rather than by the presence of gas *per se* (18). Our findings extend this concept by highlighting that biochemical markers—especially albumin and procalcitonin (PCT)—offer early prognostic information in patients managed conservatively. Hypoalbuminemia is a recognized marker of malnutrition, systemic inflammation, and capillary leak (19, 20). In HPVG, decreased oncotic pressure from low albumin fosters intestinal interstitial edema; in shock/inflammation states, capillary leakage and suppressed hepatic synthesis further aggravate hypoalbuminemia (17, 21). Edema with impaired perfusion undermines the mucosal barrier and promotes bacterial translocation and systemic inflammation (22); clinical/experimental data also suggest that restoring oncotic pressure can improve mesenteric perfusion (23). PCT is a sensitive indicator of bacterial infection and sepsis (24–26), upregulated via IL-6 and TNF-α–mediated inflammatory pathways. Markedly higher PCT in non-survivors likely reflects severe infection and multi-organ dysfunction, both key drivers of HPVG-related mortality. In addition, elevated BNP among non-survivors may indicate sepsis-related myocardial dysfunction or fluid overload, further compromising perfusion and exacerbating intestinal ischemia. Radiologically, the peripheral branching/tree-like lucencies typical of HPVG often resolve completely with conservative management; in clinically stable patients, sequential CT/ultrasound follow-up combined with PCT and albumin can support bedside risk stratification and treatment monitoring.

This study has several limitations. First, the small sample size and single-center retrospective design limited the statistical power and generalizability of this study. Because of the limited number of events, the reported associations may be unstable and susceptible to random error. Thus, our findings should be interpreted cautiously as preliminary signals that require confirmation in larger, preferably multicenter prospective studies. Second, the retrospective design precludes control of potential confounders—such as nutritional status granularity, source control of infection, and treatment delays. Third, selection bias may have influenced the relatively high in-hospital mortality observed in this study. As a retrospective single-center series of conservatively managed HPVG cases from a tertiary hospital, the cohort may have been affected by institutional case mix, regional population characteristics, local disease spectrum, and referral patterns. These factors may have contributed to enrichment of patients with more severe underlying disease or systemic deterioration. Accordingly, the mortality rate observed in this study should be interpreted cautiously and may not be directly generalizable to other centers or broader HPVG populations. Fourth, Multivariable analysis was not performed in this study. Although such analysis would be important to assess the independent prognostic value of albumin and PCT, the small sample size and limited number of death events did not support reliable adjusted modeling. Under these conditions, multivariable regression would have been prone to overfitting and unstable estimates. Therefore, the observed associations should be interpreted as exploratory and unadjusted rather than as evidence of independent prognostic effects. In addition, the small sample size and heterogeneity of underlying etiologies precluded statistically credible subgroup analyses according to specific causes of HPVG, such as intra-abdominal infection or gastrointestinal bleeding. Fifth, albumin and PCT were analyzed only on the basis of baseline measurements obtained initial clinical evaluation. Serial dynamic changes in these biomarkers were not systematically assessed in the present study. Therefore, the potential additional prognostic value of longitudinal biomarker trends could not be evaluated. In addition, although several CT features were collected, the radiologic analysis remained primarily descriptive. Because of the small sample size, heterogeneity of underlying etiologies, and lack of surgical/pathologic confirmation in this conservatively managed cohort, the prognostic relevance of specific imaging findings could not be evaluated robustly. In addition, treatment heterogeneity should be acknowledged. Although all patients received conservative management, treatment was not protocolized and was individualized according to etiology, clinical severity, and organ dysfunction. Because this retrospective cohort spanned a long period (2017–2025), detailed treatment variables—such as antibiotic selection and duration, decompression timing and duration, fluid resuscitation targets, nutritional support strategies, and albumin supplementation regimens—were not uniformly documented in a standardized manner and therefore could not be analyzed robustly. As a result, the potential influence of treatment variation on outcomes could not be fully assessed, and the observed associations between albumin, PCT, and mortality should not be interpreted as independent of treatment interventions.

## 5 Conclusion

In conservatively managed HPVG, prognosis should be interpreted in the context of the underlying etiology and the patient’s overall systemic condition. In our cohort, lower baseline albumin and higher baseline PCT were associated with in-hospital mortality, whereas HPVG itself should be viewed as an imaging sign rather than an isolated disease process. Nevertheless, because HPVG frequently accompanies serious intra-abdominal or systemic illness, its detection should prompt timely etiologic evaluation, close clinical observation, and individualized management. Further large-scale prospective studies are required to confirm these observations.

## Data Availability

The raw data supporting the conclusions of this article will be made available by the authors, without undue reservation.
